# Differential Analysis of Anthocyanins in Red and Yellow Hawthorn (*Crataegus pinnatifida*) Peel Based on Ultra-High Performance Liquid Chromatography-Electrospray Ionization Tandem Mass Spectrometry

**DOI:** 10.3390/molecules30051149

**Published:** 2025-03-03

**Authors:** Dongsheng Wang, Beibei Cheng, Liyang Yu, Guomei Yuan, Yate Ma, Jijun Zhang, Furong Lin

**Affiliations:** 1 Hebei Province Yanshan Agriculture Characteristic Industry Technology Research Institute, Hebei Normal University of Science and Technology, Qinhuangdao 066004, China; 2 Hebei Key Laboratory of Horticultural Germplasm Excavation and Innovative Utilization, Hebei Normal University of Science and Technology, Qinhuangdao 066600, China; 3 Hebei Higher Institute Application Technology Research and Development Center of Horticultural Plant Biological Breeding, Qinhuangdao 066004, China; 4 State Key Laboratory of Tree Genetics and Breeding, Key Laboratory of Tree Breeding and Cultivation of State Forestry and Grassland Administration, Research Institute of Forestry, Chinese Academy of Forestry, Beijing 100091, China

**Keywords:** hawthorn, anthocyanins, peel, UHPLC-ESI-MS/MS

## Abstract

Anthocyanins constitute the primary pigment components in hawthorn (*Crataegus pinnatifida*) peel, yet their specific composition and concentration profiles remain poorly characterized. This study employed ultra-performance liquid chromatography–electrospray ionization–tandem mass spectrometry (UPLC-ESI-MS/MS)-based metabolomics to systematically compare anthocyanin profiles between red-peel (CPR) and yellow-peel (CPY) hawthorn cultivars. Our analysis identified 26 anthocyanin metabolites in CPR and 24 in CPY, with cyanidin-3-O-galactoside and cyanidin-3-O-arabinoside being the predominant compounds in both. Multivariate analysis revealed seven significantly differential metabolites, including cyanidin-3-O-galactoside, cyanidin-3-O-arabinoside, pelargonidin-3-O-galactoside, pelargonidin-3-O-glucoside, pelargonidin-3-O-arabinoside, and peonidin-3-O-galactoside. Notably, all the differential metabolites exhibited reductions in CPY compared to CPR. Chromatic analysis demonstrated that CPR possessed highly significantly lower hue angle values (h_ab_) than CPY (47.7093 ± 4.1706, 83.6427 ± 1.4604, *p* < 0.01), showing strong negative correlations with key anthocyanins. These findings enhance the scientific understanding of anthocyanin biosynthesis in hawthorn peel and provide a certain reference for the development and utilization of anthocyanins in hawthorn peel.

## 1. Introduction

The genus *Crataegus* (Rosaceae), commonly known as hawthorn, comprises approximately 1000 species distributed across Europe, North America, and East Asia [1]. This genus has gained recognition as a valuable source for natural pigment extraction and antioxidant-rich functional food development [2]. Among the 12 species and six varieties native to China, *C. pinnatifida* Bge. var. major N.E.Br. (Chinese hawthorn) dominates commercial cultivation. While primarily consumed fresh or processed, its peel exhibits significant anthocyanidin accumulation, with most cultivars displaying red pigmentation and rare occurrences of yellow/orange phenotypes [3,4].

Anthocyanidins, the glycosylated precursors of anthocyanins, encompass six major aglycones: cyanidin, delphinidin, peonidin, pelargonidin, malvidin, and petunidin [5,6]. These water-soluble pigments typically conjugate with glucose, arabinose, or galactose via O-glycosidic bonds, conferring both chromatic properties (cyanidin/peonidin: purple-red; pelargonidin: red-orange; delphinidin/malvidin/petunidin: blue-purple) and potent antioxidant capacities [7]. Despite their pivotal role in peel pigmentation, previous phytochemical analyses of Chinese hawthorn have prioritized organic acids, triterpenoids, and flavonoids [8,9,10], with limited attention to anthocyanin profiling. Current knowledge remains restricted to red-peel cultivars, while the anthocyanin composition of yellow-peel variants persists as a scientific enigma.

Modern metabolomics employs chromatography–mass spectrometry platforms to achieve high-resolution metabolite profiling, combining separation efficiency, analytical precision, and environmental sustainability [11,12,13]. One anthocyanin, cyanidin-3-O-galactoside, was identified when Liu et al. [14] analyzed the phenolic compounds in Chinese hawthorn fruits using HPLC-DAD-ESI/MS (high-performance liquid chromatography–diode array detector–mass spectrometry). Moreover, Wang, et al. [15] isolated and identified cyanidin-3-O-β-galactoside and cyanidin-3-O-α-arabinoside of *C. pinnatifida* Bge. var. major fruits. Subsequently, cyanidin-3-O-galactoside and cyanidin-3-O-arabinoside were extracted from Chinese hawthorn peel and analyzed using HPLC-MS [16]. Eight anthocyanins were separated and identified in the fruit of *C. pinnatifida* Bge. var. major; this is by far the most complete study of anthocyanins in the species [17]. Wang et al. [18] revealed two additional derivatives—cyanidin-3-xyloside and cyanidin-3-O-glucoside—with broad-spectrum metabolomics at five developmental stages. Ultra-high-performance liquid chromatography–tandem mass spectrometry (UHPLC-MS/MS) is becoming increasingly widely used in the analysis of metabolites [19,20].

Notwithstanding these advancements, the systematic profiling of hawthorn peel anthocyanins—particularly comparative analyses between chromatic phenotypes—remains unaddressed. This study employs UPLC-ESI-MS/MS-based metabolomics to qualitatively and quantitatively analyze the major anthocyanins of red- and yellow-skinned *C. pinnatifida* Bge. var. major and to identify phenotype-specific differential anthocyanins. Our findings are helpful in revealing the reason for the coloration of hawthorn peel and in understanding the mechanism of the color change of hawthorn peel.

## 2. Results

### 2.1. Quality Control (QC) Analysis of Samples

The total ion chromatogram (TIC) profiles exhibited comprehensive ion intensity distribution across the analytical time course (Figure S1a). A mixed solution was used as the quality control (QC) sample; the QC sample was inserted every 10 test samples during the analysis process. High overlap of the QC-derived TIC curves (Figure S1b) confirmed excellent system stability and method reproducibility. The target anthocyanins showed well-resolved peaks in extracted ion chromatograms (Figure S1c). The method validation demonstrated limits of detection (LOD) and quantification (LOQ) ranging from 0.1909 to 0.5342 ng/g and 0.6592 to 1.8445 ng/g, respectively, meeting the stringent sensitivity requirements for plant metabolite analysis.

### 2.2. Qualitative and Quantitative Analysis of Anthocyanin

A total of 26 anthocyanin metabolites in six categories were detected in CPR and CPY, including cyanidin derivatives (n = 4), delphinidin derivatives (n = 6), malvidin derivatives (n = 2), pelargonidin derivatives (n = 6), peonidin derivatives (n = 4), and petunidin derivatives (n = 4) (Table 1). Ten compounds were authenticated using reference standards: cyanidin-3-O-galactoside, cyanidin-3-O-arabinoside, cyanidin-3-O-(6-O-malonyl-β-D-glucoside), delphinidin-3-O-galactoside, malvidin-3-O-arabinoside, malvidin-3-O-galactoside, pelargonidin-3-O-glucoside, peonidin-3-O-glucoside, peonidin-3-O-arabinoside, and petunidin-3-O-glucoside. The remaining compounds were identified through spectral library matching (Maiwei Biotechnology Co., Ltd., Wuhan, China). The CPR-exclusive metabolites included pelargonidin-3-O-glucoside and pelargonidin-3-O-arabinoside. The total anthocyanin content differed significantly between CPR (2411.76 μg/g) and CPY (53.967 μg/g), with cyanidin-3-O-galactoside (45.3-fold higher in CPR) and cyanidin-3-O-arabinoside (51.0-fold higher) being predominant in both cultivars.

### 2.3. Principal Component Analysis

**Figure 1:**
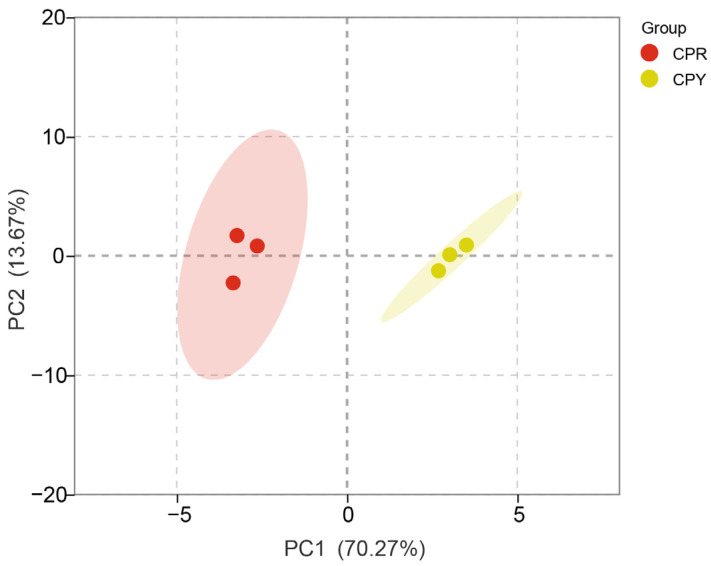
PCA scores plot of CPR and CPY. The shaded area represents the 95% confidence interval.

Principal component analysis (PCA) is an unsupervised discriminant method that can evaluate the clustering relationship of red-peel (CPR) and yellow-peel (CPY) hawthorn samples. PCA analysis was performed on the anthocyanin metabolites of CPR and CPY (Figure 1). The samples in the same group were clustered together, and there were great differences between CPR and CPY, indicating that the samples within the group had good repeatability, the data of the samples were reliable, and the subsequent differential metabolite analysis could be performed. The scores of PC1 and PC2 were 70.27% and 13.67%, respectively. This result showed that the representative features of most metabolites were well aggregated.

### 2.4. OPLS-DA Analysis and Permutation Test Analysis

**Figure 2:**
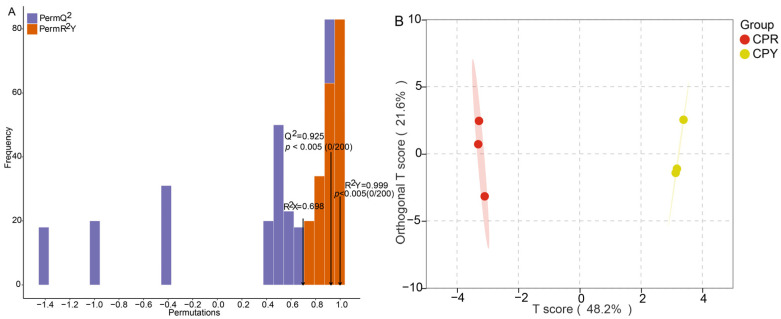
OPLS-DA analysis of CPR and CPY ((**A**) OPLS-DA verification plot of CPR and CPY; (**B**) OPLS-DA score plot of CPR and CPY, tthe shaded area represents the 95% confidence interval; ordinate and abscissa represent the scores of orthogonal and principal components in the OSC process, respectively).

**Figure 3:**
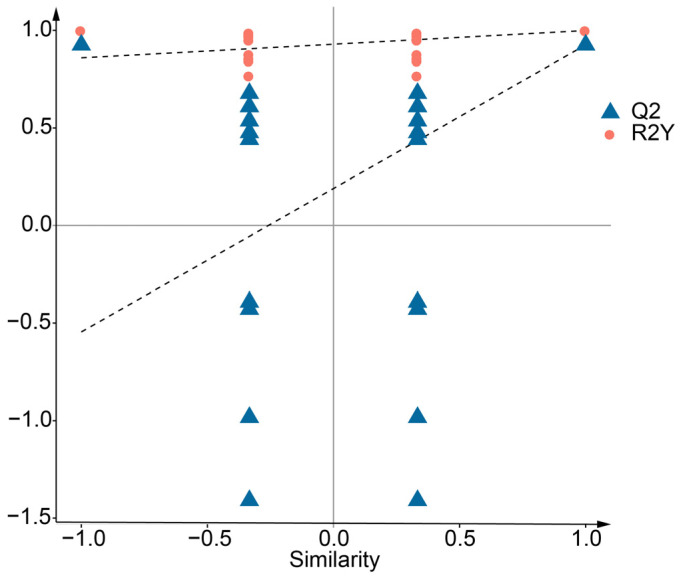
OPLS-DA permutation test of CPR and CPY. The X-axis represents the retention of permutation, while the Y-axis displays the values of R^2^Y and Q^2^. The two dashed lines correspond to the regression lines of R^2^Y and Q^2^, respectively.

Orthogonal partial least squares discriminant analysis (OPLS-DA) was also used to show the difference between CPR and CPY and to further screen differential metabolites. The results showed that the parameters R2X and R2Y of the OPLS-DA model were 0.698 and 0.999, respectively (Figure 2A), which indicated that the interpretation rate of the model was good. Q2 was 0.925, indicating that the prediction ability of the model was high. In the OPLS-DA model score plot, the T score of the principal component in the OSC (orthogonal signal correction) process was 48.2%, showing that the difference between the two groups was obvious. The orthogonal T score was 21.6%, indicating that the difference within the group was small (Figure 2B). The permutation test results showed that as the replacement retention decreased, R2 and Q2 decreased, and the regression line showed an upward trend, which indicated that the model did not have overfitting and was effective and repeatable (Figure 3).

### 2.5. Analysis of Differential Anthocyanin Metabolites

Differential anthocyanin metabolites were screened according to the following criteria: VIP (variable importance in project value) ≥ 1, *p*-value ≤ 0.05, and fold change (FC) ≥ 2 or fold change ≤ 0.5. Cyanidin-3-O-galactoside, cyanidin-3-O-arabinoside, pelargonidin-3-O-galactoside, pelargonidin-3-O-glucoside, pelargonidin-3-arabinoside, peonidin-3-O-galactoside and peonidin-3-O-arabinoside were identified as differential metabolites. Compared with CPR, all the differential metabolites in CPY showed a downward trend (Table 2).

### 2.6. Correlation Analysis Between Anthocyanin Differential Metabolites and Hue Angle

CPR displayed significantly lower hue angles (47.7093 ± 4.1706°) compared to CPY (83.6427 ± 1.4604°, *p* < 0.01), correlating with visual phenotypes (Table 3). Strong negative correlations (r = −0.9023 to −0.9955) between hue angle and differential anthocyanin content were observed (Table 4), confirming that anthocyanin accumulation drives red pigmentation intensity.

## 3. Discussion

Traditional approaches for identifying bioactive components in *Crataegus* species have primarily relied on HPLC systems coupled with detectors such as DAD, MS, and photodiode arrays (PAD) [21,22,23,24]. Anthocyanins, which are critical pigments responsible for the coloration of hawthorn fruit peel, remain under-characterized in these studies, with significant discrepancies existing across reported identifications. For instance, Zorzi et al. [21] identified delphinidin-hexoside in *Crataegus monogyna* Jacq. using HPLC-DAD-ESI-HRMS, while Simirgiotis [23] detected four anthocyanin derivatives in the same species via HPLC-DAD-MS. To date, the most comprehensive profiling was achieved using HPLC-DAD-ESI/MS in *C. pinnatifida* Bge. var. *major* N.E.Br., revealing eight distinct anthocyanins [17], the highest number reported to date.

UHPLC-ESI-MS/MS has emerged as a cornerstone technology for targeted metabolomic profiling, particularly in fruit phytochemical characterization [18,25,26]. Recent applications of this platform include the identification of 17 anthocyanins in *Kadsura coccinea* (Lem.) A. C. Smith [27] and 26 distinct anthocyanin derivatives in cashew apple (*Anacardium occidentale*) [28]. Aligning with these advances, our LC-MS analysis revealed 26 anthocyanin metabolites in *C. pinnatifida* red-fruited varieties (CPR) and 24 in their yellow-fruited counterparts (CPY). Notably, 24 anthocyanins were common to both varieties, with pelargonidin-3-O-glucoside and pelargonidin-3-O-arabinoside being uniquely detected in CPR.

Previous studies on *C. pinnatifida* have identified diverse anthocyanin profiles. Liu et al. [14] quantified cyanidin-3-O-galactoside in red hawthorn dry weight (0.06–0.66 mg/g), while Liu et al. [17] isolated eight anthocyanins, including cyanidin-3,5-dihexoside, pelargonidin-3-rutinoside, cyanidin-3-galactoside, cyanidin-3-glucoside, cyanidin-3-rutinoside, cyanidin-3-arabinoside, pelargonidin-3-glucoside, and malvidin-3-glucoside. Recent work by Wang et al. [18] further confirmed cyanidin-3-xyloside and cyanidin-3-O-glucoside. Some anthocyanins were also identified in other hawthorn fruits. *C. monogyna* Jacq. contains acylated derivatives, such as malvidin-3-O-(4′′′coumaroyl)-rutinose-5-O-glucose [23], while *C. monogyna* fruits exhibit cyanidin-O-hexoxide and cyanidin-3-O-glucoside and peonidin-O-hexoxide [24]. Notably, this study represents the first comprehensive anthocyanin analysis of yellow Chinese hawthorn, revealing only two undetected anthocyanins compared to the red varieties. Furthermore, delphinidin and petunidin derivatives were newly identified in Chinese hawthorn.

Cyanidin-3-O-galactoside emerged as the predominant anthocyanin in both CPR and CPY, constituting 98.2% and 96.7% of total anthocyanins when combined with cyanidin-3-O-arabinoside. This aligns with Liu et al.’s [17] findings in *C. pinnatifida* and parallels observations in red-skinned *C. monogyna*, where cyanidin-3-O-glucoside predominates [24]. Anthocyanin absence in yellow *C. azarolus* [29] and distinct profiles in black-skinned *C. maximowiczii* [30] further underscore the species-specific patterns. The cyanidin dominance (80% total anthocyanins) in apples [31] suggests conserved metabolic pathways across Rosaceae.

Quantitative analysis revealed that CPR’s total anthocyanin content (TAC) exceeded that of CPY by 44.69-fold, which is consistent with Qi et al.’s [32] report of ≥20-fold differences between red and yellow hawthorn cultivars. It has been reported that the reduction in anthocyanins leads to a change in the color of the flowers from coral to pink, then to yellow [33,34]. The positive values of a* indicate red, and the positive values of b* indicate yellow. For hue angle, zero degrees (0°) represents red and 90° represents yellow [35,36]. Thus, this TAC disparity directly correlates with peel coloration, as evidenced by the colorimetric parameters: CPR exhibited 4.43× higher a* (redness) and 1.83× lower b* (yellowness) versus CPY. Hue angle (h_ab_) values further confirmed phenotypic differences (CPR: 47.7 vs. CPY: 83.6), with significant negative correlations between h_ab_ and differential anthocyanin metabolites (Table 4). These findings align with apricot coloration studies [37] and anthocyanin degradation patterns in strawberries [38].

Multivariate analysis (PCA/OPLS-DA) effectively distinguished CPR and CPY metabolomes (Figure 1 and Figure 2B). The screening criteria were slightly different in the studies. Differential metabolites were mined by *p* < 0.05 and VIP > 1 [39]. In another report, *p* ≤ 0.05 and fold change ≥ 2 were applied [40]. Differential metabolites were screened using stringent criteria (VIP ≥ 1, *p* ≤ 0.05, FC ≤ 0.5), identifying seven key anthocyanins: cyanidin-3-O-galactoside, cyanidin-3-O-arabinoside, pelargonidin-3-O-galactoside, pelargonidin-3-O-glucoside, pelargonid-in-3-O-arabinoside, peonidin-3-O-galactoside, and peonidin-3-O-arabinoside. All the metabolites showed reduced accumulation in CPY. Notably, despite cyanidin-3-O-galactoside predominance in CPY, its chromatic expression may be masked by co-occurring flavonoids [35,41] through co-pigmentation effects. Additional factors, including pH fluctuations, metal ion interactions [42], and environmental conditions [43], likely contribute to coloration differences, highlighting the need for process/storage condition studies.

## 4. Materials and Methods

### 4.1. Plant Materials and Color Parameters Determination

**Figure 4:**
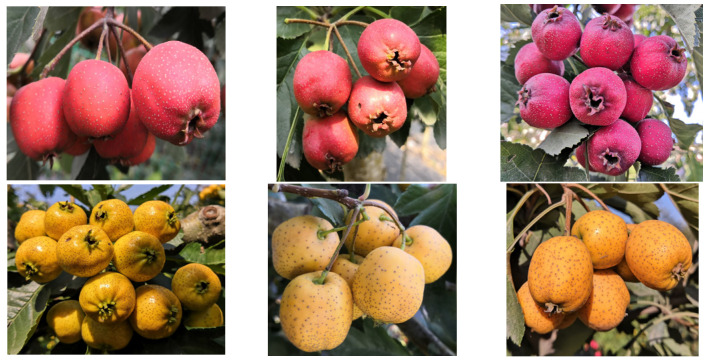
Phenotypic characterization of CPR (red peel) and CPY (yellow peel) accessions. **Upper** panel (**left** to **right**): CPR1, CPR2, CPR3; **lower** panel (**left** to **right**): CPY1, CPY2, CPY3.

Six elite hybrid progeny with red peel (designated CPR1, CPR2, CPR3) and yellow peel (CPY1, CPY2, CPY3) were selected from crosses between *C. pinnatifida* Bge. var. major ‘Damianqiu’ and *C. pinnatifida* Bge. var. major ‘Dahuangmianzha’ (Figure 4). These seedlings were uniformly grafted onto ‘Damianqiu’ rootstocks in 2017 and initiated fruiting by 2021. All the grafted plants were cultivated in identical plots under standardized horticultural management. At 140 days after anthesis in 2022, ten fully illuminated mature fruits were harvested from the upper canopy of each tree for color parameter analysis and anthocyanin quantification. Five fruits per tree were subjected to color measurements, with three technical replicates per fruit. The remaining five fruits per plant were pooled for anthocyanin extraction.

Peel color indices (a* and b*) at the equatorial region were recorded using an X-Rite SP60 portable spectrophotometer under ambient natural illumination. Hue angle (h_ab_) was calculated as h_ab_ = arctan (b*/a*) [44].

### 4.2. Extraction of Hawthorn Peel Anthocyanin

The fruits were peeled and frozen in liquid nitrogen for the determination of anthocyanin metabolites. First, freeze-dried peel was ground into powder by a Mixer mill mm 400 (30 Hz, 1.5 min, Retsch, Haan, Germany); then, 50mg of peel powder was dissolved in 500 μL of extracting solution (methanol/water/hydrochloric acid = 500:500:1 (*V*/*V*/*V*)). The extract was sonicated at 4 °C for 5 min (KQ5200E, Kunshan Shumei, Kunshan, China) and centrifuged at 12,000 rpm for 3 min (5424R, Eppendorf, Hamburg, Germany). The supernatant was filtered through 0.22 μm nylon membranes (Doudian, Shenzhen, China) prior to LC-MS/MS analysis.

### 4.3. UHPLC and ESI-MS/MS Conditions

The extracts were detected and analyzed using an UHPLC-ESI-MS/MS system (UPLC, ExionLC™ AD, Framingham, MA, USA; MS, SCIEX OTRAP 6500+, Framingham, MA, USA). An Acquity beh C18 chromatographic column (1.7 µm, 2.1 mm × 100 mm) was used. Ultrapure water containing 0.1% formic acid (Sigma-Aldrich, St Louis, MO, USA) and methanol with 0.1% formic acid (Merck, Darmstadt, Germany) were used as mobile phases A and B, respectively. Elution gradient: At 0.00 min, the proportion of phase B was 5%, increased to 50% at 6.00 min, further increased to 95% at 12.00 min, maintained for 2 min, decreased to 5% at 14 min, and equilibrated for 2 min. The column temperature was 40 °C, and the injection volume was 2 μL.

ESI+ was used as an ion source with a positive ion mode; the temperature was set at 550 °C, the ion spray voltage (IS) was 5500 V, and the curtain gas was 35 psi. Anthocyanins were quantified via multiple reaction monitoring (MRM), with optimized declustering potentials (DP) and collision energies (CE) for target ion pairs (Table 1). Parent ions were fragmented in the collision cell, and characteristic product ions were selected for quantification.

### 4.4. Anthocyanin Content Calculation and Statistical Analysis

There were 11 standards in total, including cyanidin-3-O-galactoside, cyanidin-3-O-arabinoside, cyanidin-3-O-(6-O-malonyl-beta-D-glucoside), delphinidin-3-O-galactoside, malvidin-3-O-arabinoside, malvidin-3-O-galactoside, pelargonidin-3-O-glucoside, peonidin-3-O-glucoside, peonidin-3-O-arabinoside, petunidin-3-O-glucoside, and delphinidin-3,5-O-diglucoside. All the standards (isoReag, Shanghai, China) were first prepared into a 1mg/mL stock solution with 50% methanol and stored at −20 °C. Before analysis, the working solution was diluted with 50% methanol to 0.01 ng/mL, 0.02 ng/mL, 0.05 ng/mL, 0.1 ng/mL, 0.5 ng/mL, 1 ng/mL, 5 ng/mL, 10 ng/mL, 50 ng/mL, 100 ng/mL, 500 ng/mL, 1000 ng/mL, 2000 ng/mL, and 5000 ng/mL. Then, the standard curve was drawn with the standard concentration as the abscissa and the peak area as the ordinate. With reference to the retention time of the standard, the mass spectrometry data were qualitatively analyzed based on the self-built database (Maiwei Biotechnology Co., Ltd., Wuhan, China). Analyst 1.6.3 (Sciex, Framingham, MA, USA) was used for data analysis and, MultiQuant 3.0.3 (Sciex) further performed integral correction on the mass spectrometry peaks to ensure qualitative and quantitative accuracy. Afterwards, it was combined with the standard curve, and the peak area of the component was used to obtain the concentration; then, the formula for quantitative analysis was as follows: (μg/g) = C × V/1,000,000/m. (C: concentration value (μg/mL); V: the volume of the solution used for extraction (mL); m: sample mass (g)).

The limit of quantification (LOQ) and the limit of detection (LOD) were calculated using the standard. Their formulas were LOD = kσ and LOQ = 10σ [45], respectively. The standards were prepared into a 1ng/mL solution, and detection was repeated 10 times. Then, the standard deviation (σ) of the actual measured concentration was calculated. According to the degree of freedom (n) = 9 and *p* (1) = 0.01 in the *t*-test critical value table, the k value was 2.821.

Principal component analysis (PCA) and orthogonal partial least-squares discriminant analysis (OPLS-DA) were implemented using Stats 3.5.1 and Metabo Analyst R 1.0.1 in R 3.5.1. Model validity was confirmed through 200 permutation tests. VIP, *p*-value, and fold change were combined to select the differential metabolites.

## 5. Conclusions

A total of 26 anthocyanin metabolites were identified through UHPLC-ESI-MS/MS analysis, with complete detection (26/26) in CPR and partial detection (24/26) in CPY. Both accessions exhibited identical dominant anthocyanin profiles, with cyanidin-3-O-galactoside and cyanidin-3-O-arabinoside constituting the most abundant components in peel tissues. Comparative analysis revealed seven differentially accumulated metabolites: cyanidin-3-O-galactoside, cyanidin-3-O-arabinoside, pelargonidin-3-O-galactoside, pelargonidin-3-O-glucoside, pelargonidin-3-O-arabinoside, and peonidin-3-O-galactoside. Notably, all the differential metabolites displayed downregulation in CPY compared to CPR. This study expands the documented anthocyanin repertoire of *C*. *pinnatifida* fruits. However, comprehensive characterization of hawthorn peel anthocyanins requires further investigation with expanded sample sizes and enhanced metabolite coverage.

## Figures and Tables

**Table 1:** Absolute quantification of anthocyanin results.

Anthocyanin Metabolite	Retention Time	Regression Equation	Precursor Ions *m*/*z*	Characteristic Fragment *m*/*z*	DP	CE	CPR (μg/g)	CPY (μg/g)	Limits of Detection (ng/g)	Limits of Quantification (ng/g)
Cyanidin-3-O-galactoside	6.91	y = 5424.31085x + 1862.34714(0.99881)	449.1	287.1	50	32	2327.962 ± 435.075 ^Aa^	51.406 ± 30.260 ^Bb^	0.1909	0.6592
Cyanidin-3-O-arabinoside	7.87	y = 2.06939 × 10^5^x + 8.27387 × 10^4^ (0.99727)	419.1	287.1	45	25	40.410 ± 10.411 ^Aa^	0.792 ± 0.225 ^Bb^	0.3996	1.3798
Cyanidin-3-O-(6-O-malonyl-beta-D-glucoside)	10.20	y = 1.42967 × 10^5^x + 23953.76856(0.99900)	535.1	287.1	100	32	0.007 ± 0.003 ^Aa^	0.004 ± 0.002 ^Aa^	0.2292	0.8291
Cyanidin-3-(6-O-p-caffeoyl)-glucoside	11.38	y = 3.62073 × 10^4^x + 4600.17059(0.99612)	611.1	287.1	75	35	0.013 ± 0.004 ^Aa^	0.009 ± 0.008 ^Aa^	-	-
Delphinidin-3-O-arabinoside	6.96	y = 3.62073 × 10^4^x + 4600.17059(0.99612)	435.5	303.1	100	30	0.038 ± 0.022 ^Aa^	0.046 ± 0.009 ^Aa^	-	-
Delphinidin-3-O-(6-O-p-coumaroyl)-glucoside	11.75	y = 3.62073 × 10^4^x + 4600.17059(0.99612)	611.1	303.1	75	35	0.026 ± 0.008 ^Aa^	0.033 ± 0.030 ^Aa^	-	-
Delphinidin-3-O-(6-O-acetyl)-glucoside	10.97	y = 3.62073 × 10^4^x + 4600.17059(0.99612)	507.1	303.1	75	35	0.180 ± 0.006 ^Aa^	0.094 ± 0.093 ^Aa^	-	-
Delphinidin-3-O-(6-O-malonyl-beta-D-glucoside)	9.49	y = 3.62073 × 10^4^x + 4600.17059(0.99612)	551.1	303.1	100	32	1.240 ± 0.832 ^Aa^	0.216 ± 0.217 ^Aa^	-	-
Delphinidin-3-O-5-O-(6-O-coumaroyl)-diglucoside	10.93	y = 3.62073×10^4^x + 4600.17059(0.99612)	773.2	303.1	75	35	0.030 ± 0.005 ^Aa^	0.027 ± 0.016 ^Aa^	-	-
Delphinidin-3-O-galactoside	6.01	y = 1.32162 × 10^5^x − 5432.25388(0.99888)	465.1	303.1	60	31	0.332 ± 0.156 ^Aa^	0.098 ± 0.030 ^Aa^	0.4700	1.6230
Malvidin-3-O-arabinoside	9.77	y = 1.52996 × 10^5^x + 1279.51381(0.99863)	463.3	331.1	120	27	0.161 ± 0.092 ^Aa^	0.054 ± 0.028 ^Aa^	0.2055	0.7095
Malvidin-3-O-galactoside	8.95	y = 2.38083 × 10^5^x + 8974.76971(0.99770)	493.2	331.1	50	34	0.505 ± 0.310 ^Aa^	0.272 ± 0.246 ^Aa^	0.3676	1.2693
Pelargonidin-3-O-(6-O-p-coumaroyl)-glucoside	12.53	y = 3.62073×10^4^x + 4600.17059(0.99612)	579.2	271.1	75	35	0.122 ± 0.035 ^Aa^	0.105 ± 0.020 ^Aa^	-	-
Pelargonidin-3-O-5-O-(6-O-coumaroyl)-diglucoside	12.04	y = 3.62073 × 10^4^x + 4600.17059(0.99612)	741.2	271.1	75	35	0.026 ± 0.003 ^Aa^	0.025 ± 0.004 ^Aa^	-	-
Pelargonidin-3-O-galactoside	7.78	y = 3.62073 × 10^4^x + 4600.17059(0.99612)	433.2	271.1	100	27	20.269 ± 8.060 ^Aa^	0.194 ± 0.091 ^Ab^	-	-
Pelargonidin-3-O-rutinoside-5-O-glucoside	8.92	y = 3.62073 × 10^4^x + 4600.17059(0.99612)	741.2	271.1	75	35	0.036 ± 0.013 ^Aa^	0.037 ± 0.005 ^Aa^	-	-
Pelargonidin-3-O-glucoside	8.37	y = 1.74307 × 10^5^x + 12033.58043(0.99738)	433.2	271.1	100	27	0.115 ± 0.025 ^Aa^	-^Bb^	0.2673	0.9474
Pelargonidin-3-O-arabinoside	8.74	y = 3.62073×10^4^x + 4600.17059(0.99612)	403.1	271.1	45	25	0.214 ± 0.0380 ^Aa^	-^Bb^	-	-
Peonidin-3-O-(6-O-p-coumaroyl)-glucoside	12.60	y = 3.62073 × 10^4^x + 4600.17059(0.99612)	609.2	301.1	75	35	0.017 ± 0.0010 ^Aa^	0.019 ± 0.003 ^Aa^	-	-
Peonidin-3-O-glucoside	8.96	y = 3.26812 × 10^5^x + 1.63886 × 10^5^ (0.99506)	463.3	301.1	100	30	0.093 ± 0.040 ^Aa^	0.015 ± 0.003 ^Ab^	0.5342	1.8445
Peonidin-3-O-galactoside	8.49	y = 3.62073 × 10^4^x + 4600.17059(0.99612)	463.3	287.1	100	27	19.245 ± 2.193 ^Aa^	0.316 ± 0.015 ^Bb^	-	-
Peonidin-3-O-arabinoside	9.37	y = 2.31273 × 10^5^x + 8.07477 × 10^4^ (0.99769)	433.2	287.1	50	25	0.220 ± 0.036 ^Aa^	0.004 ± 0.002 ^Bb^	0.3559	1.2290
Petunidin-3-O-sophoroside	7.96	y = 3.62073 × 10^4^x + 4600.17059(0.99612)	641.1	287.1	70	40	0.043 ± 0.023 ^Aa^	0.014 ± 0.013 ^Aa^	-	-
Petunidin-3-O-glucoside	8.05	y = 1.76301 × 10^5^x + 17143.60875(0.99872)	479.1	287.1	100	30	0.024 ± 0.012 ^Aa^	0.009 ± 0.008 ^Aa^	0.2225	0.7888
Petunidin-3-O-galactoside	7.98	y = 3.62073 × 10^4^x + 4600.17059(0.99612)	479.1	303.1	100	30	0.129 ± 0.0623 ^Aa^	0.047 ± 0.044 ^Aa^	-	-
Petunidin-3-O-arabinoside	8.52	y = 3.62073 × 10^4^x + 4600.17059(0.99612)	449.1	303.1	45	25	0.303 ± 0.180 ^Aa^	0.135 ± 0.027 ^Aa^	-	-
Total							2411.760 ± 369.029 ^Aa^	53.967 ± 24.424 ^Bb^		

Note: ‘-’ indicates that these compounds were not detected. Different capital letters represent highly significant differences between groups (*p* < 0.01); different lowercase letters represent significant differences between groups (*p* < 0.05).

**Table 2:** Analysis of differential anthocyanin metabolites between CPY and CPR.

Differential Anthocyanin Metabolites	VIP	*p*-Value	CPY vs. CPR Fold Change	Type
Cyanidin-3-O-galactoside	1.5141	0.0117	0.0221	down
Cyanidin-3-O-arabinoside	1.4857	0.0222	0.0196	down
Pelargonidin-3-O-galactoside	1.4037	0.0497	0.0095	down
Pelargonidin-3-O-glucoside	1.5081	0.0159	0.0000	down
Pelargonidin-3-O-arabinoside	1.5236	0.0103	0.0000	down
Peonidin-3-O-galactoside	1.5342	0.0044	0.0164	down
Peonidin-3-O-arabinoside	1.5196	0.0088	0.0174	down

**Table 3:** Calculation of a*, b*, and hue angle.

	a*	b*	h_ab_
CPR	29.1311 ± 2.6221 ^A^	32.4199 ± 2.7185 ^B^	47.7093 ± 4.1706 ^B^
CPY	6.5816 ± 1.6875 ^B^	59.4201 ± 2.9749 ^A^	83.6427 ± 1.4604 ^A^

Note: Different capital letters represent highly significant differences between CPR and CPY (*p* < 0.01).

**Table 4:** Correlation analysis between anthocyanin differential metabolites and hue angle in CPR and CPY.

Color Index	Differential Anthocyanin Metabolites	Correlation	*p*-Value
Hue angle	Cyanidin-3-O-galactoside	−0.9747	0.0010
Hue angle	Cyanidin-3-O-arabinoside	−0.9512	0.0035
Hue angle	Pelargonidin-3-O-galactoside	−0.9577	0.0026
Hue angle	Pelargonidin-3-O-glucoside	−0.9023	0.0139
Hue angle	Pelargonidin-3-O-arabinoside	−0.9259	0.0080
Hue angle	Peonidin-3-O-galactoside	−0.9918	0.0001
Hue angle	Peonidin-3-O-arabinoside	−0.99550	0.00003

## Data Availability

The datasets used and/or analyzed during the current study are available from the corresponding author on reasonable request.

## Supplementary Materials

The following supporting information can be downloaded at: https://www.mdpi.com/article/10.3390/molecules30051149/s1, Figure S1: Ion chromatogram. (a). The total ion chromatogram (TIC) of quality control (QC) sample. (b). The overlap diagram of the TIC plot from QC mass spectrometry at different times. (c). The multipeak detection plot shows the extracted ion chromatogram of anthocyanins. The abscissa represents the retention time (Time, min), and the ordinate represents the ion current intensity (Intensity, cps).
