# Supplementary material for: Differential Analysis of Anthocyanins in Red and Yellow Hawthorn (Crataegus pinnatifida) Peel Based on Ultra-High Performance Liquid Chromatography-Electrospray Ionization Tandem Mass Spectrometry
Source: Molecules. 2025 Mar 3;30(5):1149. doi: 10.3390/molecules30051149 (PMC11901954; doi:10.3390/molecules30051149)
Supplement: Supplementary file 1 [file molecules-30-01149-s001.zip › Supplementary File/Figure S1 abstract.pdf]

Figure S1. Ion chromatogram. a. The total ion chromatogram (TIC). b. The overlap diagram of the TIC plot from QC mass spectrometry at different times. c. The extracted ion chromatogram of anthocyanins. The abscissa represents the retention time (Time, min), and the ordinate represents the ion current intensity (Intensity, cps).
