# Supplementary figures and images for: Differential Analysis of Anthocyanins in Red and Yellow Hawthorn (Crataegus pinnatifida) Peel Based on Ultra-High Performance Liquid Chromatography-Electrospray Ionization Tandem Mass Spectrometry
Source: Molecules. 2025 Mar 3;30(5):1149. doi: 10.3390/molecules30051149 (PMC11901954; doi:10.3390/molecules30051149)

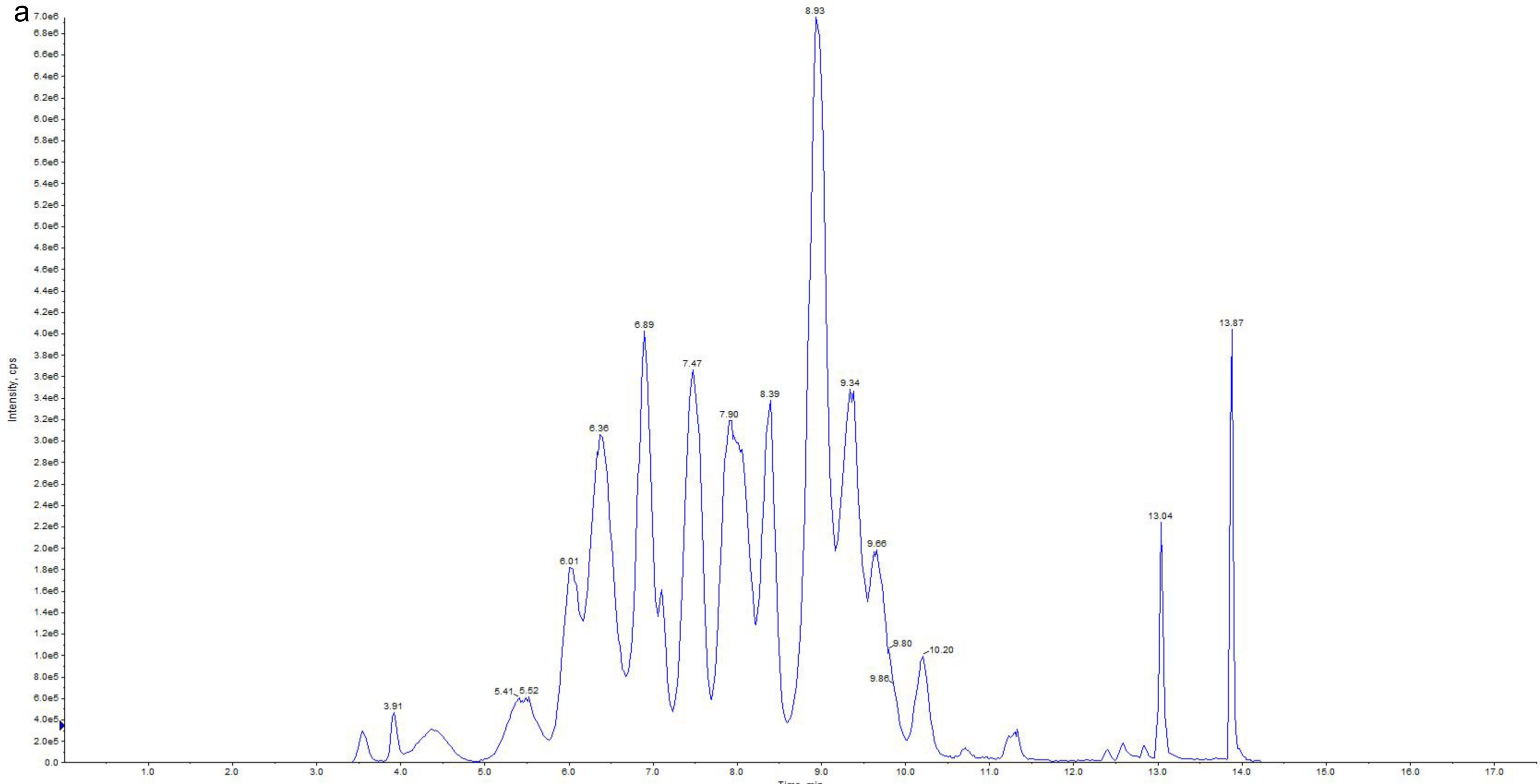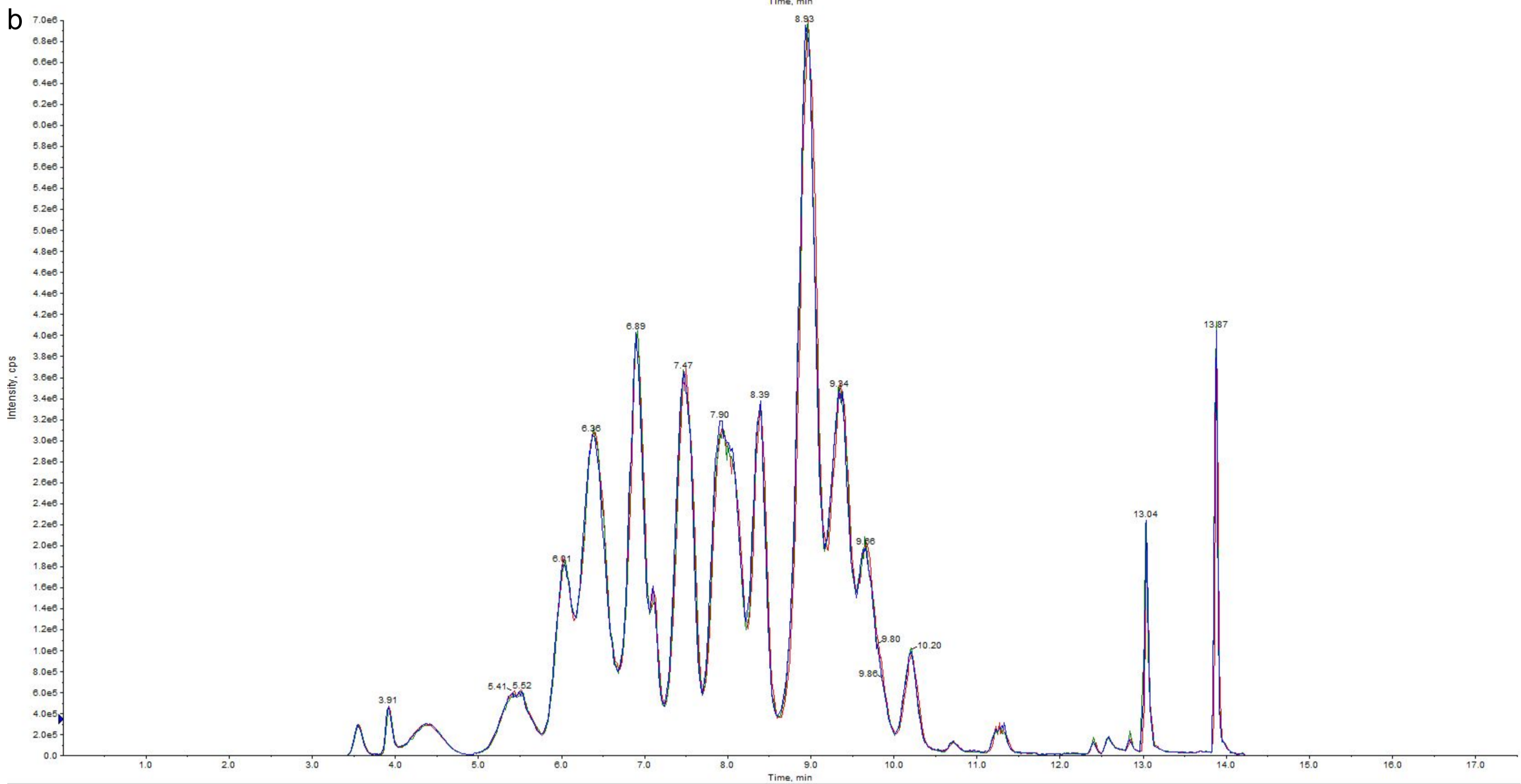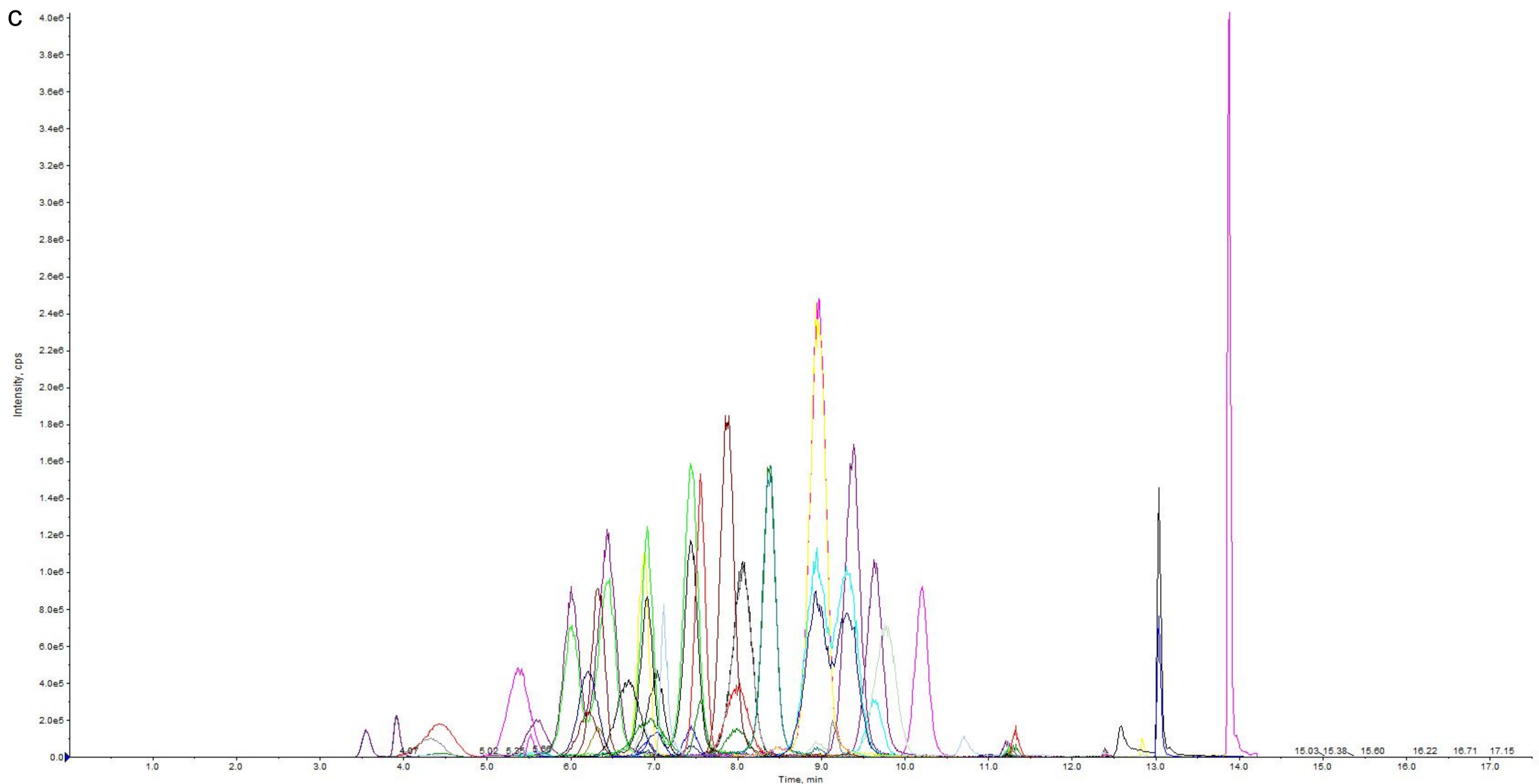

Supplement: Supplementary file 1 [file molecules-30-01149-s001.zip › Supplementary File/Figure S1. Ion chromatogram.pdf]
